# Computational Fluid Dynamics Modeling of Symptomatic Intracranial Atherosclerosis May Predict Risk of Stroke Recurrence

**DOI:** 10.1371/journal.pone.0097531

**Published:** 2014-05-12

**Authors:** Xinyi Leng, Fabien Scalzo, Hing Lung Ip, Mark Johnson, Albert K. Fong, Florence S. Y. Fan, Xiangyan Chen, Yannie O. Y. Soo, Zhongrong Miao, Liping Liu, Edward Feldmann, Thomas W. H. Leung, David S. Liebeskind, Ka Sing Wong

**Affiliations:** 1 Department of Medicine and Therapeutics, the Chinese University of Hong Kong, Prince of Wales Hospital, Shatin, Hong Kong SAR, China; 2 UCLA Stroke Center, Los Angeles, California, United States of America; 3 Department of Neurology, Beijing Tiantan Hospital, Capital Medical University, Beijing, China; 4 Department of Neurology, Tufts University, Boston, Massachusetts, United States of America; University Medical Center (UMC) Utrecht, Netherlands

## Abstract

**Background:**

Patients with symptomatic intracranial atherosclerosis (ICAS) of ≥70% luminal stenosis are at high risk of stroke recurrence. We aimed to evaluate the relationships between hemodynamics of ICAS revealed by computational fluid dynamics (CFD) models and risk of stroke recurrence in this patient subset.

**Methods:**

Patients with a symptomatic ICAS lesion of 70–99% luminal stenosis were screened and enrolled in this study. CFD models were reconstructed based on baseline computed tomographic angiography (CTA) source images, to reveal hemodynamics of the qualifying symptomatic ICAS lesions. Change of pressures across a lesion was represented by the ratio of post- and pre-stenotic pressures. Change of shear strain rates (SSR) across a lesion was represented by the ratio of SSRs at the stenotic throat and proximal normal vessel segment, similar for the change of flow velocities. Patients were followed up for 1 year.

**Results:**

Overall, 32 patients (median age 65; 59.4% males) were recruited. The median pressure, SSR and velocity ratios for the ICAS lesions were 0.40 (−2.46–0.79), 4.5 (2.2–20.6), and 7.4 (5.2–12.5), respectively. SSR ratio (hazard ratio [HR] 1.027; 95% confidence interval [CI], 1.004–1.051; P = 0.023) and velocity ratio (HR 1.029; 95% CI, 1.002–1.056; P = 0.035) were significantly related to recurrent territorial ischemic stroke within 1 year by univariate Cox regression, respectively with the c-statistics of 0.776 (95% CI, 0.594–0.903; P = 0.014) and 0.776 (95% CI, 0.594–0.903; P = 0.002) in receiver operating characteristic analysis.

**Conclusions:**

Hemodynamics of ICAS on CFD models reconstructed from routinely obtained CTA images may predict subsequent stroke recurrence in patients with a symptomatic ICAS lesion of 70–99% luminal stenosis.

## Introduction

Patients with ischemic stroke or transient ischemic attack (TIA) due to intracranial atherosclerosis (ICAS) face a high risk of stroke recurrence, especially in lesions with 70–99% luminal stenosis, which are commonly considered as anatomically severe lesions [1]–[3]. An ICAS lesion responsible for an ischemic stroke or TIA is commonly termed as symptomatic ICAS. According to the Warfarin versus Aspirin for Symptomatic Intracranial Disease (WASID) trial and the Stenting and Aggressive Medical Management for Preventing Recurrent Stroke in Intracranial Stenosis (SAMMPRIS) trial, those with symptomatic ICAS of 70–99% luminal stenosis could face a risk of recurrent stroke up to 20% at 1 year, despite of medical or interventional therapies [2]–[4]. Thus, the severity of luminal stenosis has been regarded as an indicator to guide clinical decisions in scenarios of symptomatic ICAS or patients' selection for relevant clinical trials [5]. However, the percentage of luminal stenosis only represents one aspect of ICAS, which as a complex disease has many aspects that are probably related to prognosis of symptomatic ICAS, for instance, plaque characteristics, perfusion status downstream, and presence of collaterals [6]–[8]. Despite the increasing emphasis on hemodynamic significance of the lesion in diagnosing coronary artery disease (CAD) by cardiologists in the past decade [9], [10], hemodynamic characteristics of ICAS has been paid little attention.

Previously the feasibility of reconstructing computational fluid dynamics (CFD) models of ICAS lesions based on routinely obtained computed tomographic angiography (CTA) source images has been demonstrated. And an index termed pressure ratio has been developed to represent the change of pressures across an ICAS lesion, which was the ratio of pressures distal and proximal to the lesion [11]. Physiologically, blood pressure declines with blood flowing distally, though the pressure gradient is minimal in normal arteries [12]. Thus, the distal to proximal pressure ratio in a symptomatic ICAS lesion is theoretically constantly less than 1, with a lower pressure ratio indicating a hemodynamically more significant lesion, similar with the noninvasively simulated fractional flow reserve (FFR) for CAD [10]. Moreover, according to a previous study investigating flow patterns in intracranial atherosclerotic disease by using the CFD technique, highest shear strain rates (SSR) and blood flow velocities could be detected at the throat of stenosis [13]. It also has been demonstrated that increased shear rates in the case of arterial stenosis have a definite impact on platelet activation, adhesion and aggregation, and may play a role in destabilization of atherosclerotic plaques [14]–[18]. Therefore, in this study, we aimed to evaluate hemodynamic characteristics of ICAS as depicted by CFD models, including changes of pressure, SSR and flow velocity across the lesion, and to investigate the relationships between these hemodynamic parameters and risk of stroke recurrence, in patients receiving medical treatment for a recent ischemic stroke or TIA due to ICAS of 70–99% luminal stenosis.

## Methods

### Ethics Statement

This was a retrospective cohort study approved by the Joint Chinese University of Hong Kong-New Territories East Cluster Clinical Research Ethics Committee, Hong Kong SAR, China, which was conducted according to the principles expressed in the Declaration of Helsinki. All patients were screened and selected from a prospectively collected dataset at Prince of Wales Hospital, Hong Kong SAR, China, for which they or their legal representatives had provided written informed consent.

### Subjects

Patients with a symptomatic ICAS lesion of 70–99% luminal stenosis were screened from a prospectively collected dataset at Prince of Wales Hospital (December 2006 to February 2012), Hong Kong SAR, China, and were enrolled according to the following criteria: 1) adult ischemic stroke or TIA patients admitted within one week of ictus; 2) patient undergoing clinically indicated CTA and digital subtraction angiography (DSA) examinations of intracranial arteries; 3) the stroke or TIA was due to ICAS of 70–99% luminal stenosis diagnosed by DSA, located at M1 segment of middle cerebral artery (MCA-M1), intracranial portion of internal carotid artery (ICA), intracranial portion of vertebral artery, or basilar artery; 4) receiving routine medical treatment, for instance, antiplatelet therapy and risk factor control; 5) with 1 year regular follow-up. Those underwent interventional angioplasty of the target ICAS during hospitalization were excluded from this study.

Demographics, histories of common diseases (previously diagnosed or taking corresponding medications at baseline), characteristics of the index event (ischemic stroke or TIA), results of laboratory tests at baseline, and percentage of luminal stenosis on DSA, were retrospectively collected from clinical records and radiology reports. The percentage of cross-sectional stenosis by DSA was calculated as the percentage of reduction in the vessel cross-sectional area based on the percentage of luminal stenosis, assuming that the vessel lumen and the ICAS lesion were circular and symmetric. For instance, ICAS lesions of 70%, 80%, and 90% luminal stenosis by DSA were respectively calculated to be of 91%, 96%, and 99% cross-sectional stenosis. CTA source images were retrieved and reconstructed to CFD models for evaluation of hemodynamic characteristics of the ICAS lesions as detailed below. Follow-up data at 1 year were retrieved from the prospectively collected dataset, and retrospectively confirmed against clinical records. Primary endpoint was defined as recurrent ischemic stroke in the territory (SIT) of the qualifying artery within 1 year after the index stroke, confirmed by new ischemic lesions on follow-up brain CT or magnetic resonance imaging. Secondary endpoints included any recurrent ischemic stroke or TIA, and all-cause death.

### CFD Modeling

Routinely obtained CTA source images were retrieved from the Picture Archiving and Communication System at the hospital, which were then anonymized and stored for CFD simulation of blood flow. The arterial segment containing a target ICAS lesion was visually identified and selected on CTA images, which was then segmented using a gradient-driven level set method to extract the vascular volume [19]. Centerlines were computed from selected inlet and outlet points placed at the extreme points of the vessel segment. Vessel diameters along the centerlines were derived using the Voronoi method, which allowed construction of three-dimensional (3D) vessel geometry. A mesh of the 3D surface, composed of approximately 500,000 tetrahedral cells, was then created using ANSYS ICEM-CFD (ANSYS, Inc.). Blood flow simulation was performed on this mesh using ANSYS CFX software. Generation of mesh and simulation of blood flow were run on a Cray CX1 cluster (Cray Inc.).

The following settings and assumptions were applied in construction of CFD models and simulation of blood flow. 1) Rigid, non-compliant walls with no-slip boundary conditions were assumed, as with the assumptions applied in the limited number of preceding studies on CFD of intracranial arterial stenosis [13], [20]. 2) The inlet pressure was set to be 120 mmHg, based on previous findings that mean blood pressure proximal to occlusive lesions at terminal ICA or proximal MCA, measured during intra-arterial thrombolysis, were only slightly lower than systemic blood pressure, in lesions with various degrees of luminal stenosis [21]. 3) The mean flow velocity at the outlet was prescribed as 60 cm·s^−1^, which is a typical mean flow velocity of cerebral arteries. 4) Blood was an incompressible Newtonian fluid with a constant viscosity of 0.004 kg·m^−1^·s^−1^ and was modeled based on the Navier-Stokes equations [22]. 5) Blood had a density of 1,060 kg·m^−3^.

### Evaluation of Hemodynamics of ICAS on CFD Models

**Figure 1:**
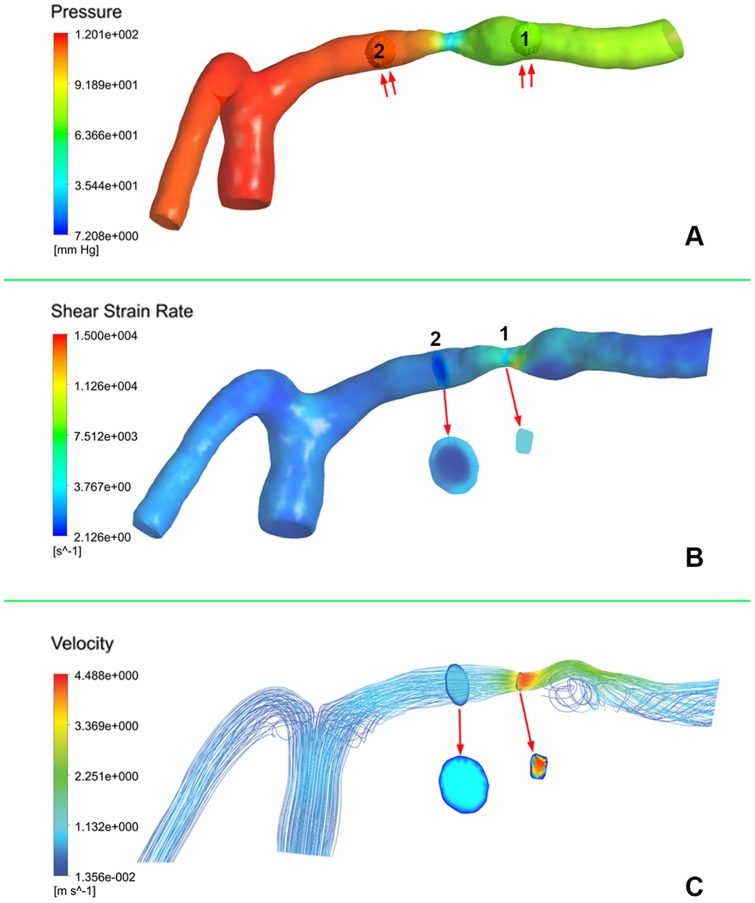
Evaluation of computational fluid dynamics model of an atherosclerotic lesion of left middle cerebral artery. A: Post- and pre-stenotic pressures were measured using spherical volumes-of-interest (VOI, double arrows) at the first anatomically normal diameters distal (VOI 1) and proximal (VOI 2) to the lesion, respectively. The pressure ratio was calculated by dividing the mean pressure at VOI 1 by the mean pressure at VOI 2. B: Shear strain rates (SSR) were respectively measured at the stenotic throat (cut-plane 1) and at the first anatomically normal diameter (cut-plane 2) proximal to the lesion, by using cut-planes perpendicular to the direction of blood flow (arrow). The SSR ratio was calculated by dividing the SSR averaged over cut-plane 1 by the SSR averaged over cut-plane 2. C: Velocities were similarly measured (arrow) as with the SSRs, and the velocity ratio was similarly calculated as with the SSR ratio.

Simulated CFD models were post-processed in ANSYS CFX-post (ANSYS, Inc.) for extraction and evaluation of hemodynamic parameters. For each ICAS lesion, changes of pressure, SSR, and flow velocity across the lesion were evaluated on the CFD model, by calculating the pressure, SSR, and velocity ratios with the following methods. The pressure ratio was calculated as the ratio of post- and pre-stenotic pressures (unit: mmHg), which were respectively measured at the 1^st^ anatomically normal diameters distal and proximal to an ICAS lesion, using spherical volumes-of-interest with the same size within the vessel domain (Figure 1A). SSR ratio of an ICAS lesion was calculated as the ratio of mean SSR (unit: s^−1^) at the stenotic throat and mean SSR at pre-stenotic vessel segment, which were respectively measured at the throat of stenosis and at the 1^st^ anatomically normal diameter proximal to the lesion, by using cut-planes perpendicular to the direction of blood flow (Figure 1B). Similar with measurement of SSRs and calculation of the SSR ratio, flow velocities (unit: m·s^−1^) were also measured at these two cut-planes and the velocity ratio was accordingly calculated (Figure 1C).

### Statistical Analyses

Demographics, medical histories, anatomic and hemodynamic characteristics of the ICAS lesions, and primary and secondary endpoint events, were described using means ± standard deviation, medians (interquartile range) or numbers (%). All variables were compared between patients with or without SIT within 1 year, by using Wilcoxon rank-sum tests or Fisher's exact tests, respectively for continuous and categorical variables. All hemodynamic indices were then analyzed in univariate Cox regression for the endpoint of SIT, as well as other variables with P<0.10 in Wilcoxon rank-sum tests or Fisher's exact tests, to calculate the hazard ratios (HR) and the 95% confidence intervals (CI) of the predictors for SIT within 1 year. For hemodynamic indices that were statistically significant in univariate Cox regression, Kaplan-Meier curves were performed to detect the cumulative probabilities of SIT based on different levels of the parameters, and receiver operating characteristic (ROC) curves and the c-statistics were performed to evaluate the predictive values of these parameters for subsequent SIT. All statistical analyses were performed using PASW Statistics (version 18.0; IBM SPSS Statistics, Chicago, U.S.A) and MedCalc (version 11.4; MedCalc Software, Ostend, Belgium). Two-sided p-values of <0.05 were considered statistically significant.

## Results

Overall, 32 patients (median age of 65; 59.4% males) with symptomatic ICAS of 70–99% luminal stenosis were recruited into this study, among whom 27 (84.4%) cases were ischemic stroke at baseline with a median National Institutes of Health Stroke Scale (NIHSS) of 2 (1–3), others being TIA. Twenty-seven (84.4%), 24 (75.0%), 13 (40.6%), 5 (15.6%) and 0 patients had histories of dyslipidemia, hypertension, diabetes, prior ischemic stroke/ TIA, and ischemic heart disease, respectively. Detailed information on demographics, medical histories and results of laboratory tests of these patients are shown in Table 1.

**Table 1:** Baseline characteristics of all patients, and patients with and without recurrent ischemic stroke in the territory of the qualifying artery.

Characteristics a	Overall (N = 32)	Recurrent ischemic stroke in the territory		P values b
		Yes (n = 6)	No (n = 26)	
Baseline characteristics				
Age, y	65 (57–72)	65 (54–70)	65 (58–74)	0.612
Male	19 (59.4)	3 (50.0)	16 (61.5)	0.666
Current smoker	15 (46.9)	3 (50.0)	12 (46.2)	1.000
History of dyslipidemia	27 (84.4)	6 (100)	21 (80.8)	0.555
History of hypertension	24 (75.0)	5 (83.3)	19 (73.1)	1.000
History of diabetes mellitus	13 (40.6)	4 (66.7)	9 (34.6)	0.194
History of ischemic stroke / TIA	5 (15.6)	1 (16.7)	4 (15.4)	1.000
Laboratory tests				
Fasting blood glucose, mmol/L	6.8±2.4	7.6 (5.3–12.9)	5.7 (5.2–7.1)	0.109
Glycosylated hemoglobin, %	6.6±1.6	7.8 (6.1–10.9)	6.1 (5.6–6.5)	0.030
Triglycerides, mmol/L	1.6±0.8	1.5 (1.1–3.1)	1.3 (1.2–1.7)	0.546
High-density lipoprotein, mmol/L	1.2±0.4	0.9 (0.8–1.4)	1.1 (1.0–1.4)	0.190
Low-density lipoprotein, mmol/L	3.4±1.3	3.5 (1.4–4.9)	3.4 (2.6–4.0)	0.981
Characteristics of ICAS				
Percentage of luminal stenosis by DSA, %	76 (70–87)	80 (71–87)	76 (70–84)	0.697
Percentage of cross-sectional stenosis by DSA, %	94 (91–98)	95 (91–98)	94 (91–97)	0.697
Hemodynamic characteristics on CFD models				
Pressure ratio	0.40 (−2.46–0.79)	−0.80 (−46.48–0.78)	0.47 (−2.28–0.80)	0.412
SSR ratio	4.5 (2.2–20.6)	22.6 (8.4–87.9)	3.5 (1.9–9.1)	0.038
Velocity ratio	7.4 (5.2–12.5)	12.1 (9.1–77.1)	6.5 (4.7–12.0)	0.038

TIA, transient ischemic attack; ICAS, intracranial atherosclerosis; DSA, digital subtraction angiography; CFD, computational fluid dynamics; SSR, shear strain rate.

a Values are means ± standard deviation, medians (interquartile range) or numbers (%).

b P values for Wilcoxon rank-sum tests or Fisher's exact tests, respectively for continuous and categorical variables.

### Anatomic and Hemodynamic Characteristics of the ICAS lesions

For the qualifying ICAS lesions evaluated in this study, 23 (71.9%), 7 (21.9%), and 2 (6.2%) lesions respectively located at MCA-M1, intracranial portion of ICA, and vertebrobasilar arteries. Anatomic and hemodynamic indices of these lesions are shown in Table 1. The median percentages of luminal stenosis and cross-sectional stenosis of these ICAS lesions were 76% (70–87%) and 94% (91–98%) by DSA, respectively. For the evaluation of reconstructed CFD models, the median values of pressure ratio was 0.40 (−2.46–0.79). The median SSRs measured at the throat of stenosis and at the 1^st^ anatomically normal diameter proximal to the lesions were 3,793 (1,888–12,570) and 948 (520–1,382) s^−1^, respectively. The median flow velocities measured at the two same cut-planes were 4.7 (2.6–9.9) and 0.7 (0.4–0.8) m·s^−1^, respectively. Median SSR ratio and velocity ratio for the 32 ICAS lesions were 4.5 (2.2–20.6) and 7.4 (5.2–12.5), respectively.

### Comparison of Characteristics between Patients with and without SIT

The primary endpoint of SIT at 1 year was identified in 6 (18.8%) patients, and secondary endpoints of any recurrent ischemic stroke or TIA, and all-cause death, occurred in 10 (31.3%) and 1 (3.1%) patients, respectively. Results for comparison of demographics and medical histories of the patients, and anatomic and hemodynamic indices of the symptomatic ICAS lesions, are shown in Table 1. For all these variables, only the baseline glycosylated hemoglobin level (P = 0.030), and the SSR ratio (P = 0.038) and velocity ratio (P = 0.038) of ICAS on the CFD models, were significantly different between patients with and without the primary endpoint of SIT. However, the percentages of luminal stenosis (80% versus 76%; P = 0.697), or cross-sectional stenosis (95% versus 94%, P = 0.697) by DSA, were not significantly different between those with or without SIT. Demographics, medical histories, and results of other laboratory tests, did not significantly differ between the two groups, either.

### Predictors of SIT

Results of univariate Cox regression for predictors of SIT in these patients are shown in Table 2. Elevated baseline glycosylated hemoglobin level (HR 1.475; 95% CI, 1.116–1.950; P = 0.006), and higher SSR ratio (HR 1.027; 95% CI, 1.004–1.051; P = 0.023) and velocity ratio (HR 1.029; 95% CI, 1.002–1.056; P = 0.035) of ICAS on the CFD models, were found to be significantly correlated to recurrent ischemic stroke in the territory of the qualifying artery at 1 year. Lower pressure ratio of ICAS on CFD models tended to be related to higher risk of subsequent SIT, but not statistically significant (HR 0.984; 95% CI, 0.966–1.002; P = 0.074).

**Table 2:** Results of univariate Cox regression for predictors of recurrent ischemic stroke in the territory of the qualifying artery.

Characteristics	HR (95% CI)	P values
Glycosylated hemoglobin, %	1.475 (1.116–1.950)	0.006
Hemodynamic characteristics on CFD models		
Pressure ratio	0.984 (0.966–1.002)	0.074
SSR ratio	1.027 (1.004–1.051)	0.023
Velocity ratio	1.029 (1.002–1.056)	0.035

HR, hazard ratio; CI, confidence interval; SSR, shear strain rate.

**Figure 2:**
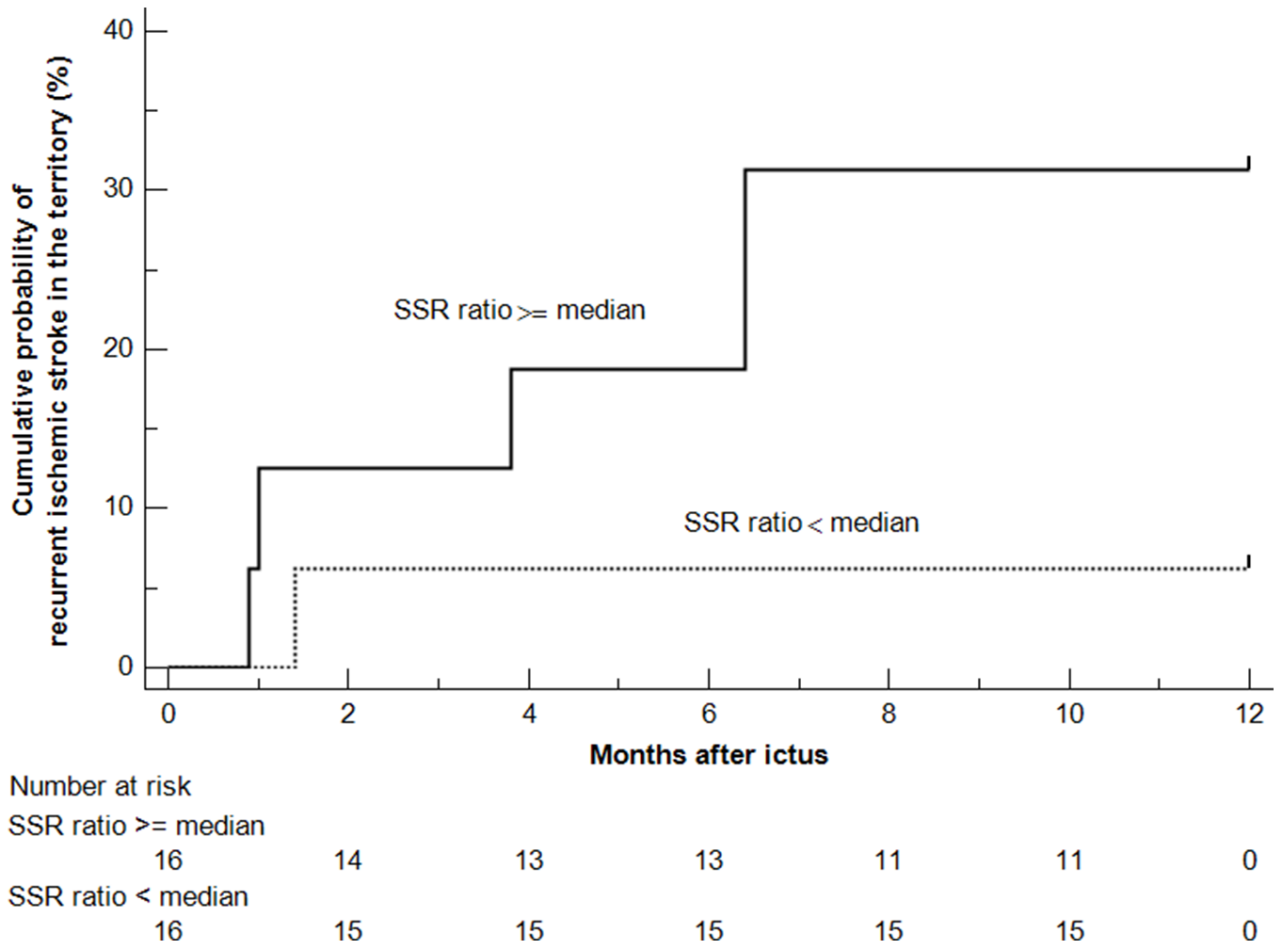
Kaplan-Meier curves for the cumulative probabilities of recurrent ischemic stroke in the territory (SIT) of the stenotic artery within 1 year after ictus, according to the shear strain rate (SSR) ratio (≥ or < median) as evaluated on the computational fluid dynamics models. Intracranial atherosclerotic lesions with a SSR ratio of ≥ median may relate to a higher risk of SIT, compared with lesions with a SSR ratio of < median (HR 5.483; 95% CI, 1.105–27.211; log-rank P = 0.079).

**Figure 3:**
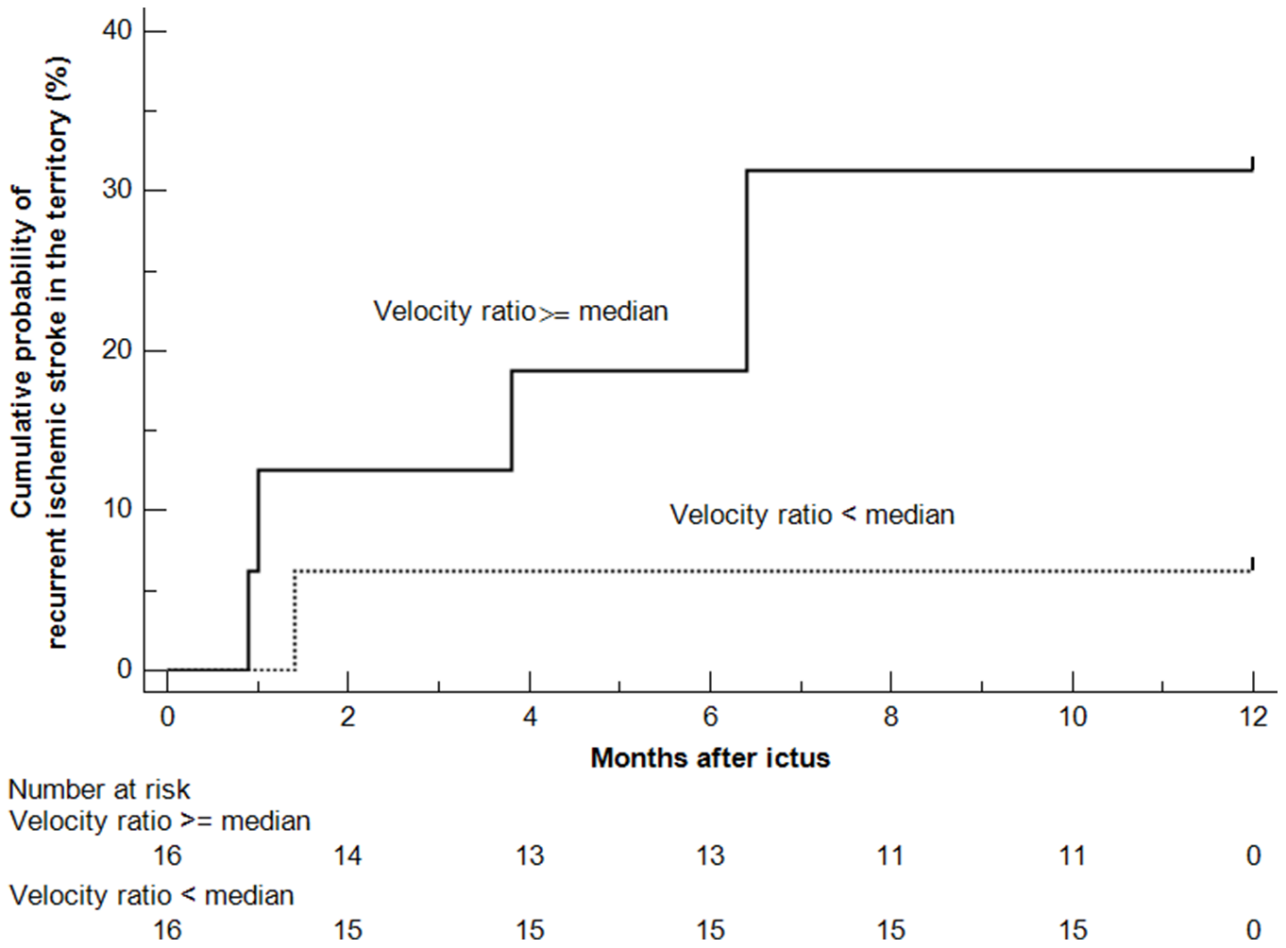
Kaplan-Meier curves for the cumulative probabilities of recurrent ischemic stroke in the territory (SIT) of the stenotic artery within 1 year after ictus, according to the velocity ratio (≥ or < median) as evaluated on the computational fluid dynamics models. Intracranial atherosclerotic lesions with a velocity ratio of ≥ median may relate to a higher risk of SIT, compared with lesions with a velocity ratio of < median (HR 5.483; 95% CI, 1.105–27.211; log-rank P = 0.079).

**Figure 4:**
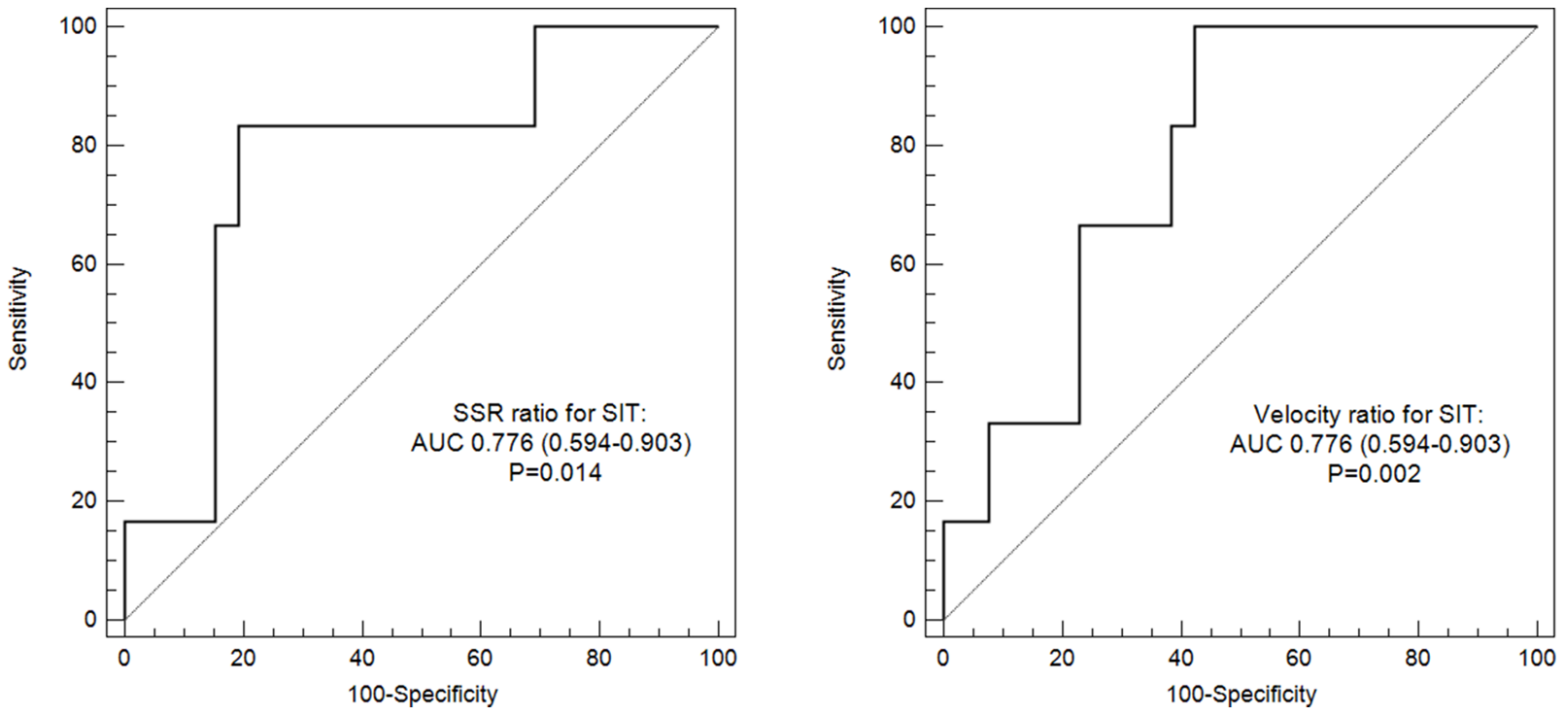
Receiver operating characteristic curves for shear strain rate ratio (left) and velocity ratio (right) to predict recurrent ischemic stroke in the territory of the qualifying artery. SIT indicates recurrent ischemic stroke in the territory of the qualifying artery; AUC, area under curve.

Kaplan-Meier curves for the cumulative probabilities of SIT in patients with ICAS lesions with SSR ratios of ≥ or < median are shown in Figure 2. In patients with symptomatic ICAS lesions of 70–99% luminal stenosis, those with a SSR ratio ≥ median may face a higher risk of recurrent ischemic stroke in the territory of the stenotic artery (HR 5.483; 95% CI, 1.105–27.211; log-rank P = 0.079), as compared with those with a SSR ratio < median. The same trend was found for patients with ICAS lesions with a velocity ratio of ≥ or < median (HR 5.483; 95% CI, 1.105–27.211; log-rank P = 0.079), as shown in Figure 3. ROC analyses revealed substantial predictive values of SSR ratio and velocity ratio for subsequent SIT within 1 year after an ischemic stroke or TIA due to symptomatic ICAS of 70–99% luminal stenosis, with the c-statistics of 0.776 (95% CI, 0.594–0.903; P = 0.014) and 0.776 (95% CI, 0.594–0.903; P = 0.002), respectively (Figure 4).

## Discussion

In this study, we applied the CFD technique in simulating blood flow through intracranial atherosclerotic lesions based on CTA images. Of the 32 patients with symptomatic ICAS of 70–99% luminal stenosis, 18.8% had a recurrent ischemic stroke in the territory of the target artery within 1 year after the index event, which was comparable to the WASID and SAMPPRIS trials [2]–[4], [23]. We found that the changes of shear strain rates and blood flow velocities across the lesions, as evaluated on the CFD models, were significantly related to SIT at 1 year, with substantial predictive values by ROC analyses.

The target patients of this study were those with a symptomatic ICAS lesion of 70–99% luminal stenosis, though some may argue that further risk stratification of such lesions, which have already been defined as of “high risk” in relevant clinical trials, may not be of clinical significance. However, many of these patients are probably not truly at high risk, based on the WASID and SAMPPRIS trials [1], [3], [4], and increasing evidences are suggesting that the severity of luminal stenosis is probably not the only or the most important indicator for the risk of stroke recurrence in these patients. Therefore, using a simple parameter of percent stenosis to stratify such lesions may be misleading [24]. For instance, at least in this preliminary study, neither the percentages of luminal stenosis of the ICAS lesions by DSA, nor the hence estimated severities of cross-sectional stenosis, were different between those with or without SITs. Thus, a simple measure or estimation of the severity of arterial stenosis, either luminal or cross-sectional, may not be an optimal indicator for patient selection in clinical trials investigating the effects of different treatment strategies for patients with symptomatic ICAS lesions, which may be one of the reasons to explain the negative findings in previous relevant trials. For instance, the SAMMPRIS trial did not prove the benefit of stenting over aggressive medical treatment for patients with symptomatic ICAS of 70–99% luminal stenosis [4]. Moreover, the Trial of Cilostazol in Symptomatic Intracranial Arterial Stenosis II (TOSS-2) failed to show significant difference between 2 dual antiplatelet therapies in preventing progression of ICAS and new ischemic lesions, in patients with symptomatic ICAS of mild to severe luminal stenosis [25]. Thus, in this study we tried to investigate the clinical relevance of hemodynamic characteristics of symptomatic ICAS of 70–99% luminal stenosis, which theoretically incorporated effects of all morphological features of such lesions, to bring new insights into patient selection for relevant clinical trials and risk stratification of affected patients.

The inclusion criterion for the percentage of stenosis in this study was based on DSA images, which is currently the gold standard for evaluating arterial luminal stenosis. Although biplane DSA images could also be reconstructed to CFD models [26], computational models in this study were based on CTA source images, which by themselves provide three-dimensional information. In addition, CFD simulation of hemodynamics of ICAS based on CTA is probably more generalizable for clinical or research purposes, with CTA being a non-invasive and more commonly used imaging modality, in contrast to DSA. In this study, hemodynamic characteristics of ICAS from CFD models based on CTA images were more associated with recurrent SIT at 1 year, as compared with the percentage of arterial stenosis by DSA. In future studies of this kind, we therefore could eliminate the need for grading the degree of stenosis by DSA, but only rely on CTA images to build the CFD models of symptomatic ICAS lesions, so that the noninvasively obtained CTA images, combined with post-processed CFD models, could provide not only anatomic but also hemodynamic information of ICAS lesions. Pressure ratio of an ICAS lesion in this study, was derived from the concept of FFR in coronary artery disease [12]. Since blood pressure drops through an arterial stenosis, the pressure ratio of ICAS in this study, or FFR of CAD, has a theoretical normal value of 1, with lower values indicating hemodynamically more significant lesions [12]. A trend, though not statistically significant, was found in this study that lower pressure ratio of ICAS might be related to a higher risk of subsequent SIT. Future studies with larger sample sizes, or enrolling patients with wider profiles of percent stenosis, may further verify such relationships. Minus values of distal pressure and hence minus pressure ratios in CFD models of some cases in this study, as shown in Table 1, were probably attributed to the prescribed outflow velocity or extremely low downstream flow, which could occur in simulated blood flow models but not in arteries in vivo. This was a limitation of this study that is further discussed below.

Shear strain rate of blood flow within the vessel lumen represents the spatial gradient of flow velocity or changes of flow velocity between infinitesimally thin layers of blood flow [13]. Increased flow velocity at the stenotic throat usually lead to increased SSR. The absolute values of SSR varied to a great extend among lesions with various flow patterns, so we used the ratio of SSRs measured at the stenosis throat and the proximal normal vessel segment, to represent changes of SSRs through a lesion, similarly for the use of velocity ratio. According to the results, higher velocity ratio or SSR ratio of ICAS correlated to increased risk of stroke recurrence, which may be explained by the effects of increased SSR on platelet functions [15]. Previous studies have found definite impact of increased shear rates in the case of arterial stenosis on platelet activation, adhesion and aggregation, and hence thrombus formation, through different pathways under different ranges of shear rates [14]–[16]. Moreover, endothelial shear stress (ESS) at the neck of a stenotic plaque, which is proportional to the product of local SSR and blood viscosity, may play a role in destabilization of atherosclerotic plaques [17], [18]. Further in vivo and in vitro studies are needed to validate potential mechanisms for findings in this study on the association between high SSR of ICAS and risk of stroke recurrence.

Our study has limitations. Firstly, the small sample size, and hence the small number of recurrent events in this study, did not allow multivariate analyses for adjustment of potential confounding factors, which therefore limited generalization of the study results. Some of the correlations found in this study did not reach statistical significance, which probably was also due to the small sample size. But this could be addressed in future studies with larger sample sizes. In addition, this study only enrolled patients with symptomatic ICAS of 70–99% luminal stenosis. Future studies performed in lesions with various degrees of stenosis are needed to facilitate better understanding of this disease, considering the fact that nearly half of the recurrent strokes in patients with symptomatic ICAS occurred in those with lesions of 50–69% luminal stenosis in the WASID trial [27]. Secondly, some minus distal pressure values as detected were probably due to the effects of the prescribed flow velocity at the outlet of the models in cases with minimal distal flow, which could be addressed in future studies by setting opening boundary conditions at the outlet, or by setting personalized outflow velocities based on flow velocities measured by transcranial Doppler or other methods. Moreover, a constant pressure rather than more patient-specific, physiological pulsatile pressures used as inlet boundary conditions in this study may reduce the accuracy and realisticity of the CFD models. But such effects of this generic constant inlet pressure may be mitigated, since we were using relative pressure ratios but not absolute pressure values to represent the changes of pressures across the symptomatic ICAS lesions. Thirdly, for intracranial arteries, it may not be possible to validate the pressures derived from CFD models against in vivo measurement, by floating a pressure sensor during the performance of catheter angiography, as is currently done for the assessment of coronary arterial pressures. It will arouse ethical issues, to invasively measure intracranial arterial pressures only for a diagnostic purpose while risking periprocedural complications of DSA. However, the hemodynamic impact of ICAS lesions as revealed by CFD models may be indirectly validated by comparing with findings from imaging methods capable of selectively quantifying regional cerebral blood flow, such as vessel-selective arterial spin-labeling magnetic resonance perfusion [28].

## Conclusions

Hemodynamic characteristics of ICAS, as evaluated on CFD models reconstructed from routinely obtained CTA images, may predict subsequent risk of stroke recurrence in patients with symptomatic ICAS of 70–99% luminal stenosis. Future prospective studies with larger sample sizes are warranted to further validate findings of this study, which may shed new light on risk stratification of this patient subset.
